# Prefrontal parvalbumin cells are sensitive to stress and mediate anxiety-related behaviors in female mice

**DOI:** 10.1038/s41598-019-56424-9

**Published:** 2019-12-24

**Authors:** Chloe E. Page, Ryan Shepard, Kelsey Heslin, Laurence Coutellier

**Affiliations:** 1 Department of Neuroscience, The Ohio State University, Wexner Medical Center, 333 W. 10th Ave, Columbus, OH 43210 USA; 2 Department of Psychology, The Ohio State University, 225 Psychology Building, 1835 Neil Avenue, Columbus, Ohio 43210 USA

**Keywords:** Prefrontal cortex, Stress and resilience

## Abstract

Reduced activity of the prefrontal cortex (PFC) is seen in mood disorders including depression and anxiety. The mechanisms of this hypofrontality remain unclear. Because of their specific physiological properties, parvalbumin-expressing (PV^+^) inhibitory interneurons contribute to the overall activity of the PFC. Our recent work using a chronic stress mouse model showed that stress-induced increases in prefrontal PV expression correlates with increased anxiety-like behaviors in female mice. Our goal is now to provide a causal relationship between changes in prefrontal PV^+^ cells and changes in emotional behaviors in mice. We first show that, in addition to increasing overall level of PV expression, chronic stress increases the activity of prefrontal PV^+^ cells. We then used a chemogenetic approach to mimic the effects of chronic stress and specifically increase the activity of prefrontal PV^+^ cells. We observed that chemogenetic activation of PV^+^ cells caused an overall reduction in prefrontal activity, and that chronic activation of PV^+^ cells lead to increased anxiety-related behaviors in female mice only. These results demonstrate that activity of prefrontal PV^+^ cells could represent a novel sex-specific modulator of anxiety-related behaviors, potentially through changes in overall prefrontal activity. The findings also support the idea that prefrontal PV^+^ cells are worth further investigation to better understand mood disorders that are more prevalent in female populations.

## Introduction

Emotional behaviors are regulated by interconnected brain regions including the hippocampus, amygdala and prefrontal cortex (PFC). Abnormal functioning of these regions is associated with emotional dysregulation as seen in mood disorders like depression or anxiety. For instance, defects in glutamatergic transmission, and thereby an overall lower level of prefrontal activity, has been related to depressive symptoms and anxious behaviors^1–4^. However, the sources of changes in prefrontal activity and their relationship with behavioral symptoms remain unclear. Understanding the mechanisms underlying this so-called “hypofrontality” in mood disorders must remain a priority to promote the development of efficacious and reliable pharmacological therapies.

The PFC contains a heterogeneous population of excitatory pyramidal and inhibitory GABAergic neurons that directly regulate its activity. Prefrontal GABAergic interneurons are the primary regulators of principal projection neurons’ spiking activity. Their dysregulation is likely to affect prefrontal activity and thereby, emotional behaviors. In support of this idea, rodent-based studies have demonstrated that modulation of the activity of specific sub-populations of GABAergic interneurons changes frontal activity and alters emotional behaviors. For instance, acutely blocking the activity of somatostatin-positive inhibitory neurons in the PFC of male mice increased their behavioral emotionality^5^. The effects of chronic social stress on depressive- and anxiety-like behaviors of male mice are mediated by cholecystokinin-GABA neurons that regulate PFC activity^6^. Recently, our work demonstrated that exposure to chronic mild stress increases prefrontal PV mRNA expression and the number of parvalbumin (PV^+^)-expressing interneurons, which corresponds with heightened anxiety-like behaviors in female, but not male, mice^7,8^. Because PV expression is activity-dependent^9,10^, the increase in expression we observed could reflect increased activity of PV^+^ cells after stress. PV^+^ cells provide strong, fast-spiking inhibitory signals to principle projection neurons in the PFC^11^ and contribute to healthy cognitive and emotional functioning^12^. They are integral to balancing activity levels of pyramidal cells in the PFC and therefore, increased PV-dependent GABAergic transmission could be contributing to the hypofrontality observed in stress-induced mood disorders. Surprisingly, prefrontal PV^+^ cells have not been thoroughly explored in the context of emotional dysregulations and prefrontal hypoactivity. Some work has highlighted a role of hippocampal PV^+^ cells in anxiety^13,14^, but whether PV^+^ cells in the PFC contribute also to mood disorders marked by hypofrontality is unclear. Our recent work^7,8^ strongly suggests that plasticity of prefrontal PV^+^ neurons contributes to anxiety dysregulations following stress exposure, a known risk factor to mood disorders^15–17^. Our work also indicates that the potential role played by PV^+^ cells in regulating anxiety could be modulated by biological sex^7^.

Here, we aim at determining the effect of chronic stress on level of activity of prefrontal PV^+^ cell and testing the idea that increasing the activity of these cells is sufficient to induce an anxiety-like phenotype in mice. We used a multidisciplinary approach, first utilizing immunolabelling to quantify the number of active prefrontal PV^+^ cell following chronic stress exposure. We then used a chemogenetic DREADD (designer receptor exclusively active by designer drugs) technique in male and female mice to increase the activity of prefrontal PV^+^ cells and measure the effects on emotional behaviors in the absence of stress to test whether this intervention was anxiogenic. Our findings show that the number of prefrontal PV^+^ cell expressing the neuronal marker of activity cFos is increased by chronic stress, and that DREADD-induced activation of these cells increases anxiety-related behaviors in female mice. This information provides novel insight into the neural mechanisms underlying anxiety disorders and identifies a potentially new avenue for treatment.

## Results

### Chronic stress exposure increases prefrontal PV^+^ cell activity

**Figure 1:**
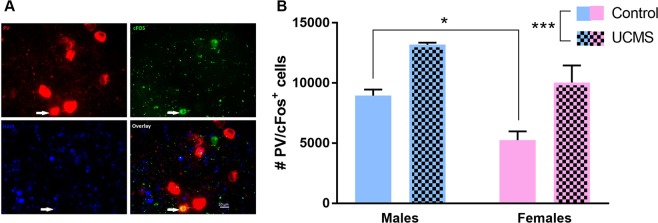
Chronic stress increases activity of prefrontal PV^+^ neurons in males and females. (**A**) Representative image showing PV (red) and cFos (green) double-labeling (white arrow) and DAPI (blue). Scale bar: 10 µm. (**B**) Chronic stress increases the number of PV/cFos double-labeled cells in the mPFC of females and males. *p = 0.05; ***p < 0.001.

We previously observed that chronic stress increases PV mRNA expression as well as the number of PV^+^ cells in the PFC of female mice^7^. We sought to confirm whether these observations indicated increased activity of this cell population by exposing male and female mice to 4 weeks of unpredictable chronic mild stress (UCMS) and measuring the number of PV^+^ cells expressing cFos, a marker of neuronal activity, in the PFC (Fig. 1). Chronic stress significantly increased the number of PV/cFos double-labeled cells in the medial PFC (mPFC) of both females and males (F_1,8_ = 28.87; p < 0.001). We also observe a significant sex effect (F_1,8_ = 16.61; p = 0.003) mostly driven by control female mice having less PV/cFos^+^ cells than control males (p = 0.02).

### Selective activation of mPFC PV^+^ cells and hypofrontality

**Figure 2:**
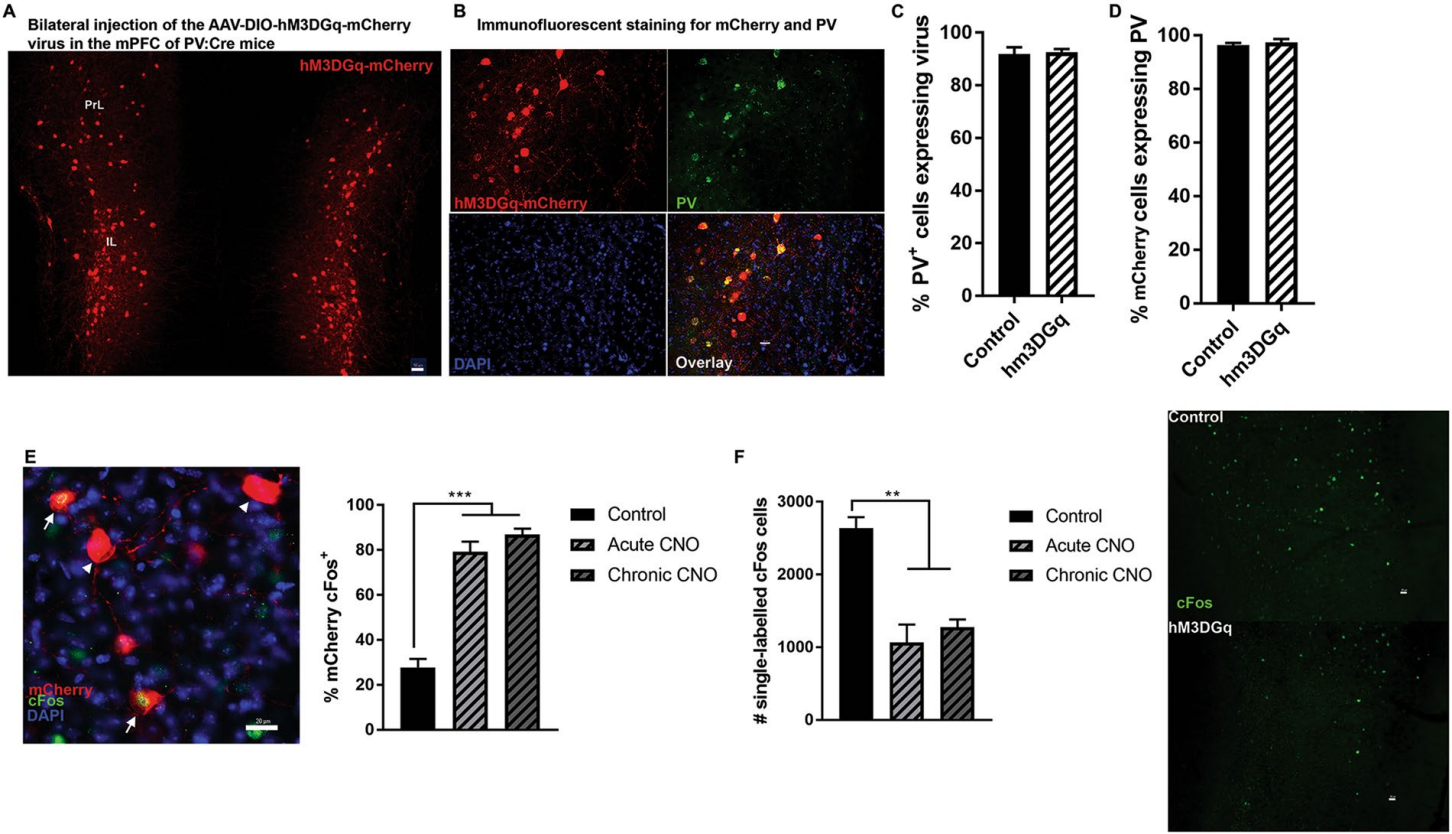
Injection of an AAV-DIO-hM3DGq DREADD virus in the mPFC of PV:Cre mice. (**A**) Representative coronal image shows bilateral expression of mCherry localized to the infralimbic cortex (IL) and part of the prelimbic cortex (PrL). Scale bar: 50 µm. (**B**) Representative images showing selective expression of the hM3DGq virus (red) in parvalbumin (PV – green) interneurons. Scale bar: 20 µm. (**C**) Stereological analysis indicated that 92.23 ± 1.31% of mPFC PV^+^ neurons expressed the control or hM3DGq virus, and (**D**) 96.85 ± 0.70% mCherry cells are PV^+^. (**E**) Verification of hM3DGq virus response to acute and chronic (once per day for 21 days) CNO injection using stereological analysis of the number of mCherry cells expressing cFos: both acute and chronic injection of CNO in the PFC of mice expressing the hM3DGq virus leads to high expression of cFos in mCherry cells when compared to cells expressing the control virus. Representative image of mCherry cells expressing cFos (white arrow) vs. mCherry cells not expressing cFos (white arrowhead). Scale bar: 20 µm. (**F**) Verification that acute and chronic chemogenetic activation of prefrontal PV^+^ cells lead to hypofunction of the PFC: both regimen of CNO injection reduce significantly the amount of cFos^+^ cells, showing overall hypofrontality. Representative images of cFos expression in the PFC of mice expressing the control or the hM3DGq virus. Scale bar: 20 µm. **p < 0.001; ***p < 0.0001.

In our chemogenetic experiments, we first verified that the DREADD virus expression is specific to PV^+^ cells. Using the unbiased stereological method in six male and female mice chosen randomly (3 mice injected with the control virus and 3 with the hm3DGq virus), we verified that 92.23 ± 1.31% of mPFC PV^+^ cells expressed the virus, and that 96.85 ± 0.70% of mCherry cells co-express PV (Fig. 2A–E). We also verified that there were no significant differences in viral expression between males and females (percentage of PV cells expressing mCherry: *p* = 0.121; percentage of mCherry cells expressing PV: *p* = 0.567).

We then verified that 0.5 mg/kg of CNO is sufficient to induce activation of PV^+^ cells after acute or chronic treatment. Acute or chronic CNO injections lead to a similar level of activity of cells expressing the hm3DGq virus: 79.23 ± 4.46% and 86.92 ± 2.64% of mCherry cells express cFos, respectively. This is significantly more than what we observed after CNO injection in mice expressing the control virus (27.68 ± 3.89%; *F*_2,5 = _61.18; *p* = 0.0003) (Fig. 2F). We finally verified that chemogenetic activation of prefrontal PV^+^ cells is sufficient to induce an overall reduction of activity of the PFC, using cFos as a neuronal marker of activity. We observed that both acute and chronic activation of PV^+^ cells reduced the number of single-labelled cFos-positive cells in the PFC 90 minutes after the acute or last CNO injection, which is significantly lower than what observed in mice expressing the control virus (*F*_2,5 = _17.44; *p* = 0.0056) (Fig. 2G). This provides indication of overall reduced activity of the PFC after chronic and acute activation of PV^+^ cells.

### Chronic activation of prefrontal PV^+^ cells is sufficient to induce an anxiety-like phenotype in female mice

**Figure 3:**
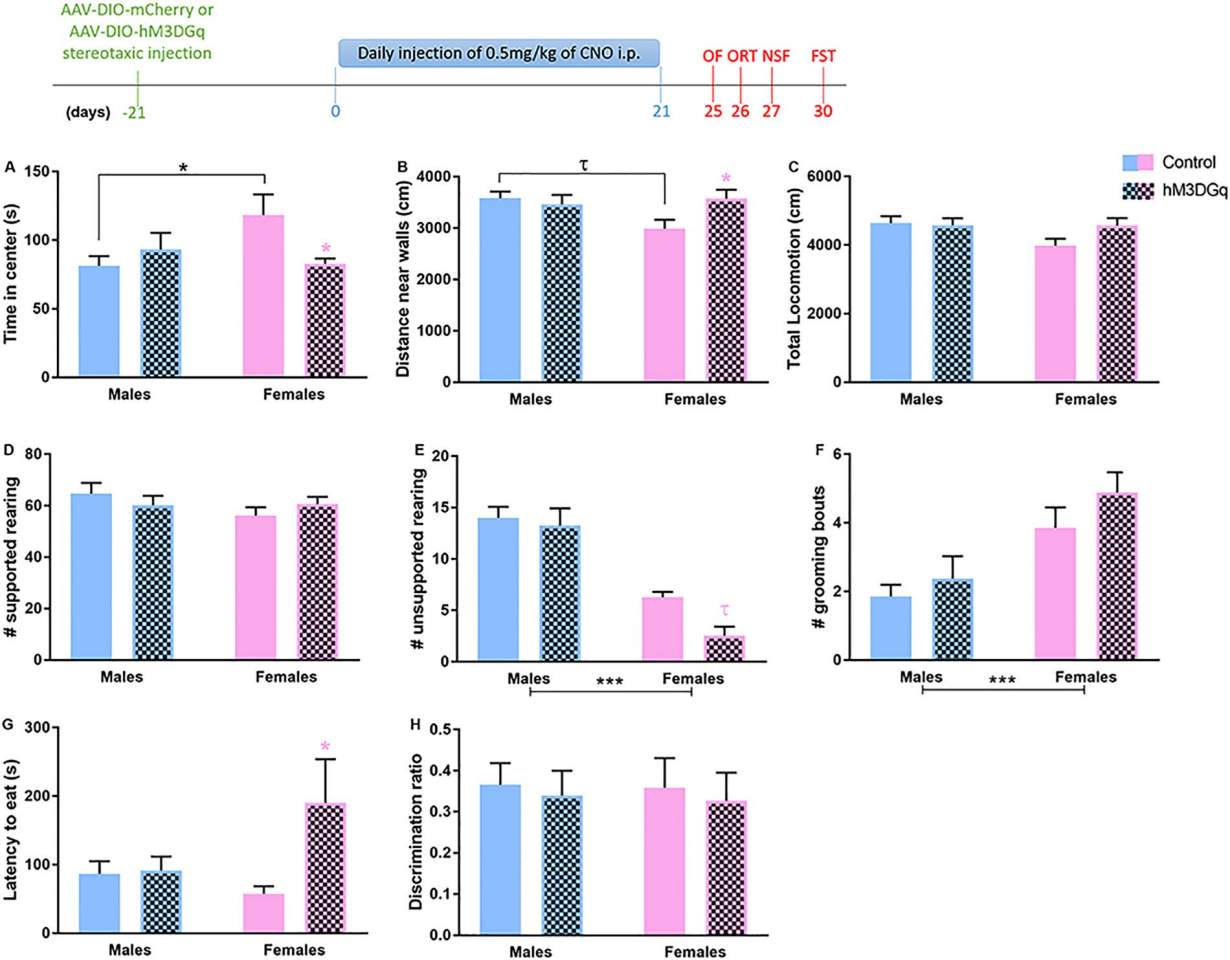
Chemogenetic chronic activation of PV^+^ neurons in the mPFC of PV:Cre male and female mice increases anxiety-like behaviors in a sex-specific manner. (Top) Experimental schematic of the timeline followed. Mice injected with the control (AAV-DIO-mCherry) or the AAV-DIO-hM3DGq virus received a daily injection of CNO over the course of 21 days. Four days after the last injection, mice were tested once daily in a behavioral test to assess various sub-domains of emotional behaviors and cognitive functions. (**A**–**F**) Behavioral endpoints measured in the OF test in control or hM3DGq mice. Female mice that underwent chronic activation of mPFC PV^+^ neurons have decreased time spent in the center of the arena, longer distance travelled near the walls, and decreased number of unsupported rearing. In males, chronic activation of mPFC PV^+^ neurons did not lead to changes in behaviors in the OF test. (**G**) Females that submitted to chronic activation of mPFC PV^+^ neurons displayed increased latency to eat the food in the NSF test, while no change in behavior was observed in males. (**H**) No effect of chronic activation of mPFC PV^+^ neurons was found in the ORT. ***p < 0.001; *p ≤ 0.05. *p = 0.057 *N* = 7–9 per group per sex. CNO: clozapine-N-oxide; OF: open field; ORT: object recognition test; NSF: novelty-suppressed feeding test.

Based on the observation that chronic stress increases the activity of prefrontal PV^+^ cells (Fig. 1), we hypothesized that exposure to daily stressors over the chronic stress period induced daily activation of PV^+^ cells. To test this idea, mice injected with the hm3DGq or control virus were treated daily with CNO over a 21-day period. We observed that overall, female mice are more sensitive to chronic activation of prefrontal PV^+^ cells than males, particularly in tests that measure anxiety-like behaviors. In the open-field (OF) test (Fig. 3A–F), we found a significant interaction between sex and virus for the time spent in the center of the arena (F_1,27_ = 5.66; p = 0.024) and distance travelled near the walls (F_1,27_ = 4.39; p = 0.045), with females with chronic activation of prefrontal PV^+^ cells spending less time in the anxiogenic center of the OF (p = 0.03), and travelling a longer path near the walls (p = 0.04). We also observed a near-significant effect of the type of virus on unsupported rearing behaviors (F_1,27_ = 3.75; p = 0.063), mostly explained by a decreased number in females exposed to chronic activation of their PV^+^ cells (p = 0.057). Similarly, in the novelty suppressed feeding test (NSF), females displayed increased sensitivity to chronic manipulation of their prefrontal PV^+^ cells (main type of virus effect: F_1,28_ = 3.94; p = 0.057; interaction: F_1,28_ = 3.41; p = 0.075; posthoc: females control vs. females hM3DGq: p = 0.02) (Fig. 3G). We did not observe a significant effect of the type of virus or interaction between sex and virus on exploratory behaviors in the OF (total locomotion and supported rearing; Fig. 3C,D), and in the object recognition test (ORT; Fig. 3H).

Interestingly, we report strong effects of sex in the OF test: control males spend less time in the center of the arena (p = 0.03) and travelled a longer path near the walls (p = 0.04) than control females. Independently of the chronic activation of PV^+^ cells, males show more unsupported rearing behaviors and less grooming than females (main sex effect: p < 0.001). These findings support increased anxiety-like behavior in males vs. females as previously reported^18,19^.

We verified that none of the effects reported could have been confounded by the chronic exposure to CNO, rather than by the chronic activation of prefrontal PV^+^ cells. Our data showed that C57Bl6/J male and female mice injected once daily with CNO for 21 days did not display differences in body weight, locomotor activity, cognitive functions and emotional behaviors when compared to vehicle injected-mice (Fig. S1).

### Acute activation of prefrontal PV^+^ cells does not induce changes in anxiety-like behaviors in females

**Figure 4:**
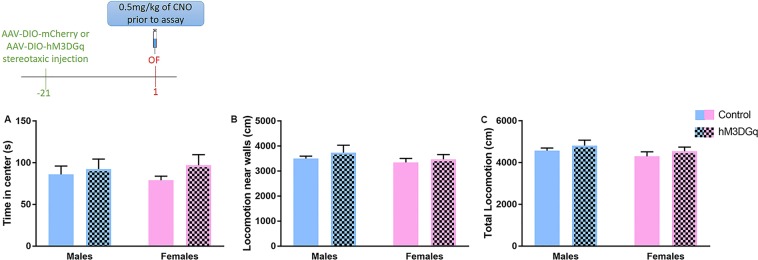
Chemogenetic acute activation of PV^+^ neurons in the mPFC of PV:Cre male and female mice does not impact behaviors in the OF test. (Top) Experimental schematic of the timeline followed. Mice injected with the control (AAV-DIO-mCherry) or the AAV-DIO-hM3DGq virus received one injection of CNO 30 minutes prior to the OF. (**A**–**C**) Acute activation of mPFC PV^+^ neurons did not impact time spent in the center, locomotion near the walls or total locomotion in the novel arena in male and female mice. *N* = 8–9 per group per sex. OF: open field.

After observing that chronic excitation of prefrontal PV^+^ cells induces anxiety-like behavior in females, we next determined whether acute activation was sufficient to elicit an anxiogenic effect. We tested a new cohort of male and female mice in the OF 30 minutes after a single CNO injection to avoid repeated PV^+^ cell activation over multiple days of behavioral testing causing chronic activation. In both females and males, we did not observe any significant effect of the type of virus, of sex, or their interaction for time spent in the center, locomotion near walls, or total locomotion in the OF arena (Fig. 4A–C). As opposed to data presented in Fig. 3, the absence of sex effect here is likely the result of the stress induced by the acute injection of CNO 30 minutes prior to testing.

Finally, we tested an additional cohort of female mice in the OF test 24 hours after a single CNO injection to examine the prolonged behavioral effects of a single activation of prefrontal PV^+^ cells. Again no significant difference between females injected with a control virus and those injected with the hM3DGq virus was noticed (Fig. S2).

## Discussion

Our findings show that the increased activity of prefrontal PV^+^ cells induced by chronic stress could be directly responsible for changes in anxiety-like behaviors, specifically in female mice. Using DREADDs to drive activity of this cell population, we mimicked the effects of chronic stress on mice anxiety-like behaviors we previously reported^7^. Together, our results establish for the first time a causal link between a chronic increase in the activity of mPFC PV^+^ cells and elevated anxiety-related behaviors in females. Further studying changes in the activity of this specific interneuronal population could be a novel avenue of research to gain a better understanding of stress-induced mood disorders, especially in the context of sex-specific differences in the occurrence and presentation of these pathologies.

Using cFos as a marker of neuronal activity, we confirmed that the previously reported chronic stress-induced increase in total PV mRNA in the PFC^7^ corresponds to increased activity of PV^+^ cells. While exposure to an anxiogenic novel arena after chronic stress increases the number of PV/cFos cells in both males and females, we observed that the effect of UCMS is more pronounced in females than in males (98% increase vs. 47%). This suggests that females prefrontal PV^+^ cells are more sensitive to stress than males. The larger change observed in females could be explained by a ceiling effect in males, as control males display higher number of PV/cFos cells than control females. This high baseline number of PV/cFos cells in males and the lower impact of 4 weeks of chronic stress could indicate that longer exposure or more severe stress is required in males to affect more profoundly prefrontal PV^+^ cells and induce an anxiety-like phenotype. This is in line with other studies reporting that male mice need a longer duration stress exposure to elicit an anxiety-like phenotype^20,21^. Alternatively, sex-specific circuits within the mPFC may also mediate anxiety-related behaviors. For example, Li *et al*. (2016) identified circuitry in the mPFC that is anxiolytic in males but not females^22^. We can speculate on a similar mechanism here, whereby a subpopulation of PV^+^ cells in the female, but not male, PFC regulates anxiety-like phenotype. It is important to note that the methodological approach used here to measure activity of PV^+^ cells provides only limited information on changes induced by stress. While it provides support for the idea that prefrontal PV^+^ cells are plastic in response to chronic stress, it fails to characterize the nature of these stress-induced changes. Others reported that chronic stress increases mIPSC in pyramidal cells and presynaptic GABA release^23^, while others showed increased dendritic arborization in GAD67^+^ prefrontal cells^24^. Future analyses of prefrontal PV^+^ cells after UCMS will help us characterize precisely chronic stress-induced changes in PV^+^ cells with regards to their physiological properties (e.g. firing rate, spike frequency, input resistance) and morphological plasticity, as well as evaluate sex differences.

Our chemogenetic manipulation of mPFC PV^+^ cell activity further supports the idea that prefrontal PV^+^ cells in female mice are more sensitive to manipulation than in males. Chronic activation of these cells reproduces the female-specific anxiogenic-like effects of chronic stress we previously reported^7,8^, while acute PV^+^ cell excitation is insufficient to induce changes in behaviors in the OF test. In both experimental manipulations, males’ behaviors remain unchanged. Again, we suspect that a ceiling effect in males contributes to our findings, as control males displayed more anxiety-like behaviors in the OF test than females, paralleling the higher level of PV/cFos cells in the male mPFC. Increasing the duration of activation of PV^+^ cells in males might be needed to induce changes in their anxiety-like behaviors. The assays we used (OF and NSF tests) are widely used to obtain measures of anxiety-like behaviors in rodent models. However, they are both based on mice disengaging from thigmotaxis behaviors. Other sub-domains of anxiety-like behaviors might not be regulated by changes in prefrontal PV^+^ cells. Previous work reported that positive allosteric modulators of GABA_A_ receptors (GABA_A_Rs) (e.g. diazepam) exert their anti-anxiety effects only when animals are tested in specific behavioral assays (e.g. elevated plus maze vs. open-field test)^25^ suggesting that the GABA system regulates only specific facets of anxiety-like behaviors. Using other tests like the elevated plus maze or the marble burying test will help address this question.

In addition, our data reveal that whether hypofrontality occurs in an acute or chronic manner can play a major role in the appearance of an abnormal phenotype. Here, we verified that increasing the activity of PV^+^ cells in the PFC leads to reduced overall prefrontal activity, as previously shown by electrophysiological recordings^26,27^. However, while both acute and chronic activation of PV^+^ cells reduce prefrontal activity, only the chronic regimen leads to increased anxiety-like behaviors in females. This finding suggests that rather than an acute state of activity of prefrontal PV^+^ cells regulating anxiety-like behaviors in females, the long-lasting changes induced by chronic activation of PV^+^ cells and their level of plasticity might better explain changes in emotional behaviors. Chronic activation of mPFC PV^+^ cells and chronic stress have both been shown to induce changes in interneuronal plasticity-related molecules and in PV^+^ neurons axonal and dendritic morphology^24,28,29^. Whether these changes within inhibitory prefrontal networks contribute directly to hypofrontality and abnormal emotional behaviors in stress-related mood disorders remains to be fully elucidated.

The present research points toward a potential novel mechanism underlying anxiety, whereby PV^+^ cell plasticity impacts the overall level of activity in the PFC leading to anxiety-like behaviors. These findings are in line with others suggesting that GABA antagonism can be anxiolytic. For example, quercetin, a negative allosteric modulator for GABA_A_Rs, reduces GABAergic transmission in the PFC^30^ and has stress-protective and anxiolytic effects^31–33^. Another negative allosteric modulator for α5-containing GABA_A_Rs, MRK-016, also exhibits antidepressant actions^34^. However, it is not known whether these compounds act on prefrontal PV^+^ cells or on PV^+^ synapses with pyramidal cells.

To conclude, the present research identifies increased prefrontal PV^+^ cell activity as a mechanism of hypofrontality as well as a sex-specific modulator of anxiety-like behaviors. Future research is necessary to determine the impact of increased prefrontal PV^+^ cell activity on downstream limbic regions that regulate anxiety behaviors, such as the amygdala and hippocampus. Further investigation is also necessary to elucidate the mechanisms by which chronic stress impacts PV^+^ cells in the PFC over the long-term, as well as the mechanisms behind sex differences in the behavioral effects of chronic PV^+^ cell activation. Overall, the findings presented here open a new avenue of research into a cell-specific mechanism potentially underlying sex differences in stress susceptibility and the prevalence of anxiety.

## Materials and Methods

### Animals

Adult male and female C57Bl/6 J mice were ordered from Jackson Laboratory (Maine, US) and were used for chronic stress exposure and evaluation of PV/cFos double-immunolabeling. They were group-housed per sex (3–5 mice per cage, unless specified otherwise) and allowed to habituate to the colony room for one week before experimental procedures began.

PV:Cre adult male and female mice (B6;129P2-Pvalbtm1(cre)Arbr/J) were used for the DREADD experiments. PV:Cre animals were bred in our colony from parents purchased from Jackson Laboratory. After weaning, mice were maintained group-housed per sex (3–5 mice per cage) and undisturbed (aside from regular cage cleaning) until experimental procedures started.

All mice had access to food and water ad libitum and were maintained on a 12-hour reverse light/dark cycle. All experiments were conducted in accordance with protocols approved by the Institutional Animal Care and Use Committee of The Ohio State University and were performed based on the National Institutes of Health Guide for the Care and Use of Laboratory Animals.

### Unpredictable chronic mild stress (UCMS) and PV/cFos immunohistochemistry

The UCMS paradigm used is the same as we previously described^7^ and consisted of single-housing the animals and of daily exposure over 4 weeks to alternating mild stressors presented in a random order according to an unpredictable schedule. Stressors included: absence of nesting material for 24 h, 20° cage tilt on the vertical axis for 6 h, absence of bedding in the cage for 8 h, restraint stress in the dark for 8 min, and restraint stress under bright light for 4 min. Control animals were handled once daily for 1–2 minutes throughout the UCMS period.

To measure activity of prefrontal PV^+^ cells in the PFC after UCMS, we used a double immunofluorescent approach for PV and cFos. Twenty-four hours after the last handling (control group) or stressor (UCMS group), mice were exposed to the open field (OF) test (see *“Behavioral testing”* section for description) to stimulate cFos induction. Ninety minutes after exposure to the open field, animals were perfused with 4% cold paraformaldehyde (PFA). Brains were removed and kept in 4% PFA at 4 °C overnight before storage in a sucrose solution (30% sucrose). Brains were frozen on dry ice and sectioned at 50 μm using a cryostat so as to obtain 3 sets of sections containing the PFC. Free-floating staining was performed on 1 set of sections using a rabbit anti-PV antibody (1:100, Abcam, Ab11427), and a goat anti-cFos antibody (1:300, SantaCruz Biotechnology, sc-52G), used as a marker of neuronal activity. Sections were then incubated with Alexa Fluor donkey anti-goat 488 and Alexa Fluor donkey anti-rabbit 555 secondary antibodies (1:500, Thermofisher A11055 and A31572, respectively). Quantitative analysis of PV^+^ cells expressing cFos (indicating activity of this specific neuronal population) in the mPFC was achieved using the unbiased stereology method with StereoInvestigator software from MBF Bioscience (Williston, VT) as previously described^7^.

### Stereotaxic viral vector injection and verification of specificity

AAV DREADD vectors were obtained from the University of North Carolina Vector Core Facilities (Chapel Hill, NC). Adult PV:Cre mice were injected bilaterally with 0.5 µl/side of AAV2/hSyn-DIO-hm3D(Gq)-mCherry (‘hm3DGq’) or with the AAV2/hSyn-DIO-mCherry (’control virus’) (~10^12^ vg/ml) into the medial PFC (mPFC – including the central part of the prelimbic (PrL) and the infralimbic (IL) cortex). Coordinates were antero-posterior +1.7 mm; medio-lateral ±0.2 mm; dorso-ventral −2.6 mm, according to the brain atlas^35^. Viruses were injected at a rate of 0.1 µl/minute. The syringe remained in place for 10 minutes before being removed. A period of 21 days was allowed to obtain full viral expression specifically within PV^+^ cells.

To verify accuracy of injection site, we collected and processed the brains of all mice after completion of all experiments. One set of PFC sections was stained using a rabbit anti-DsRed antibody (1:1000, Clontech Laboratories Inc) followed by an Alexa Fluor anti-rabbit 555 secondary antibody (1:500, Thermofisher) to target mCherry. In addition, we quantified cell-specific expression of the DREADD viral infection to PV^+^ cells. Six sets of sections from three mice injected with the control virus and three mice injected with the hm3DGq virus were selected randomly. Sections were incubated with a guinea pig anti-PV antibody (1:500, Synaptic Systems) and a rabbit anti-DsRed antibody (1:1000, Clontech Laboratories Inc). Secondary antibodies were a donkey anti guinea pig CF488A conjugate (1:500) and Alexa Fluor donkey anti-rabbit 555 (1:500). Quantification was achieved using the unbiased stereology method to count the percent of PV^+^ cells expressing mCherry, and percent of mCherry cells expressing PV.

### Pharmacogenetic activation of prefrontal PV^+^ cells

To achieve specific activation of prefrontal PV^+^ cells, we injected mice intraperitoneally with clozapine-N-oxide (CNO) prepared in 0.9% saline. Chronic activation of prefrontal PV^+^ cells was achieved through a 21-day period of daily CNO injection (0.5 mg/kg/day). Acute activation of these cells was achieved through one single injection of the same dose of CNO 30 minutes or 24 hours prior to the open field test. The CNO dose (0.5 mg/kg) was chosen based on previous work showing that it is sufficient to induce changes in firing activity of PV^+^ cells^5,13^ and to avoid chronic desensitization in our chronic activation group.

To confirm these previous reports, we used a small cohort of mice infused with the hm3DGq or the control virus in the mPFC. After 21 days, two control virus mice and three hm3DGq mice were treated acutely with a single injection of CNO (0.5 mg/kg), while three other hm3DGq-mice were treated chronically (once daily for 21 days) with CNO (0.5 mg/kg). To verify that our CNO injection leads to activation of PV^+^ cells, brains were collected 90 minutes after the acute or the last CNO injection. Sections from the PFC were double-stained to visualize mCherry (rabbit anti-DsRed antibody; 1:1000, Clontech Laboratories Inc) and cFos (goat anti-cFos; 1:300, Santa Cruz). Double-labeled mCherry/cFos cells and single-labeled cFos cells were counted using the unbiased stereology method.

### Behavioral testing

All behavioral assays were conducted during the dark phase. Mice were habituated to experimenter handling for the 3 days prior to the start of behavioral testing for 1 minute per day; on the days of testing, mice were allowed a one-hour habituation period in the testing room prior to the beginning of testing. Mice were tested in no more than one test per day with an inter-test period of 24 hours. Mice chronically injected with CNO were tested 4 days after the last CNO injection. The 4-day recovery period allowed us to avoid acute effects of CNO on our behavioral endpoints. We used *N = *8–9 mice per virus group, per sex. However, two videos of the OF were corrupted, leading to *N = *7 for the control male and female groups in OF. Both hm3DGq- and control virus-expressing mice were chronically injected with CNO. To control for a potential effect of chronic exposure to CNO on behaviors, a group of adult C57Bl6/J mice was injected with CNO or vehicle once daily over a 21-day period and then behaviorally tested (*N* = 6 per treatment per sex). Acutely-injected mice with CNO were tested in the OF test 30 minutes after the CNO injection. We used *N = *8–9 mice per virus group, per sex. A last cohort of 8 female mice was used to confirm our acute injection data, and the OF test was conducted 24 hours after the CNO injection.

#### Open field test (OF)

The OF was conducted as previously described^7^. Briefly, each mouse was placed in an unfamiliar square arena (40 × 40 cm) and were allowed to explore for a period of 10 minutes under bright light conditions. Trials were video-recorded. Videos were first analyzed using the Ethovision software XT 11.5 (Noldus) to collect conventional parameters including total distance traveled (used as indicator as general activity level) time spent, and distance travelled in the center of the OF vs. near the walls (used as measures of anxiety-like behaviors). Videos were then manually analyzed by an experimenter blind to the sex and group of the animals to collect more ethological relevant parameters including: number of self-grooming bouts, supported and unsupported rearing. Previous work showed that a decrease in unsupported (but not supported) rearing behaviors occurs after exposure to stress and is a relevant measure of anxiety-like behavior^36^.

#### Object recognition test (ORT)

Animals were tested for their long-term memory abilities in the ORT. During the learning phase, mice were placed in the OF arena with two identical unfamiliar objects that they were allowed to explore for 10 minutes. Twenty-four hours later, mice were placed back in the arena with an object from the learning phase (‘familiar object’) and a never-before met object (‘novel object’). They were again allowed to freely explore the objects for a 10-minute period. Each phase was video-recorded and video scored off-line by an experimenter blind to the group of the animals. The time spent sniffing each object during the learning and testing phase was recorded; a mouse was considered sniffing an object when its nose contacted the object or was directed at the object within 1 cm. A discrimination ratio was calculated as: (time sniffing novel object – time sniffing familiar object)/total time sniffing.

#### Novelty-suppressed feeding (NSF)

The NSF was adapted from Brachman *et al*.^37^. Mice were food restricted for a period of 24 hours (no food present in home cage). Testing took place in an unfamiliar arena (30 cm × 50 cm) under bright light conditions. The floor of the arena was covered with clean bedding. At the time of testing, a single pellet of regular chow was placed in the center of the arena on a slightly elevated platform. Each mouse was placed in a corner of the arena. The latency to feed was recorded and used as a measure of anxiety-like behavior; a maximum of 10 minutes was allowed. Mice were then returned in their home cage with the food pellet for a period of 5 minutes. The total amount of food eaten within the home cage was recorded as an indicator of motivation to feed.

### Statistical analysis

Data were analyzed using the software Prism 5.01 (GraphPad Software Inc., CA, USA). Data presented in Figs. 1, 3 and 4 were analyzed using 2-way ANOVA with sex and type of virus (control vs hM3DGq) as independent factors. When appropriate posthoc analyses were conducted. Data related to the verification of the DREADD virus expression presented in Fig. 2 were analyzed using a one-way analysis of variance (ANOVA) with type of virus (control vs hM3DGq) as the independent variable and sex as a covariate to insure no sex-specific effects of viral expression. The number of mCherry expressing cFos after acute or chronic injection of CNO (vs. control), as well as the number of single-labeled cFos cells, were analyzed using one-way ANOVA followed by Tukey’s tests for multiple comparisons. Significance was set at *p* ≤ 0.05. Data are presented as mean ± standard error of the mean (SEM).

## Supplementary information

Supplementary information

## Data Availability

The datasets generated during and/or analyzed during the current study are available from the corresponding author on reasonable request.
