# Supplementary material for: Prefrontal parvalbumin cells are sensitive to stress and mediate anxiety-related behaviors in female mice
Source: Sci Rep. 2019 Dec 24;9:19772. doi: 10.1038/s41598-019-56424-9 (PMC6930291; doi:10.1038/s41598-019-56424-9)
Supplement: Supplementary file 1 — Supplementary information [file 41598_2019_56424_MOESM1_ESM.pdf]

Supplemental information

Prefrontal parvalbumin cells are sensitive to stress and mediate anxiety-related behaviors in female mice

Chloe E Page; Ryan Shepard; Kelsey Heslin; Laurence Coutellier

Figure S1

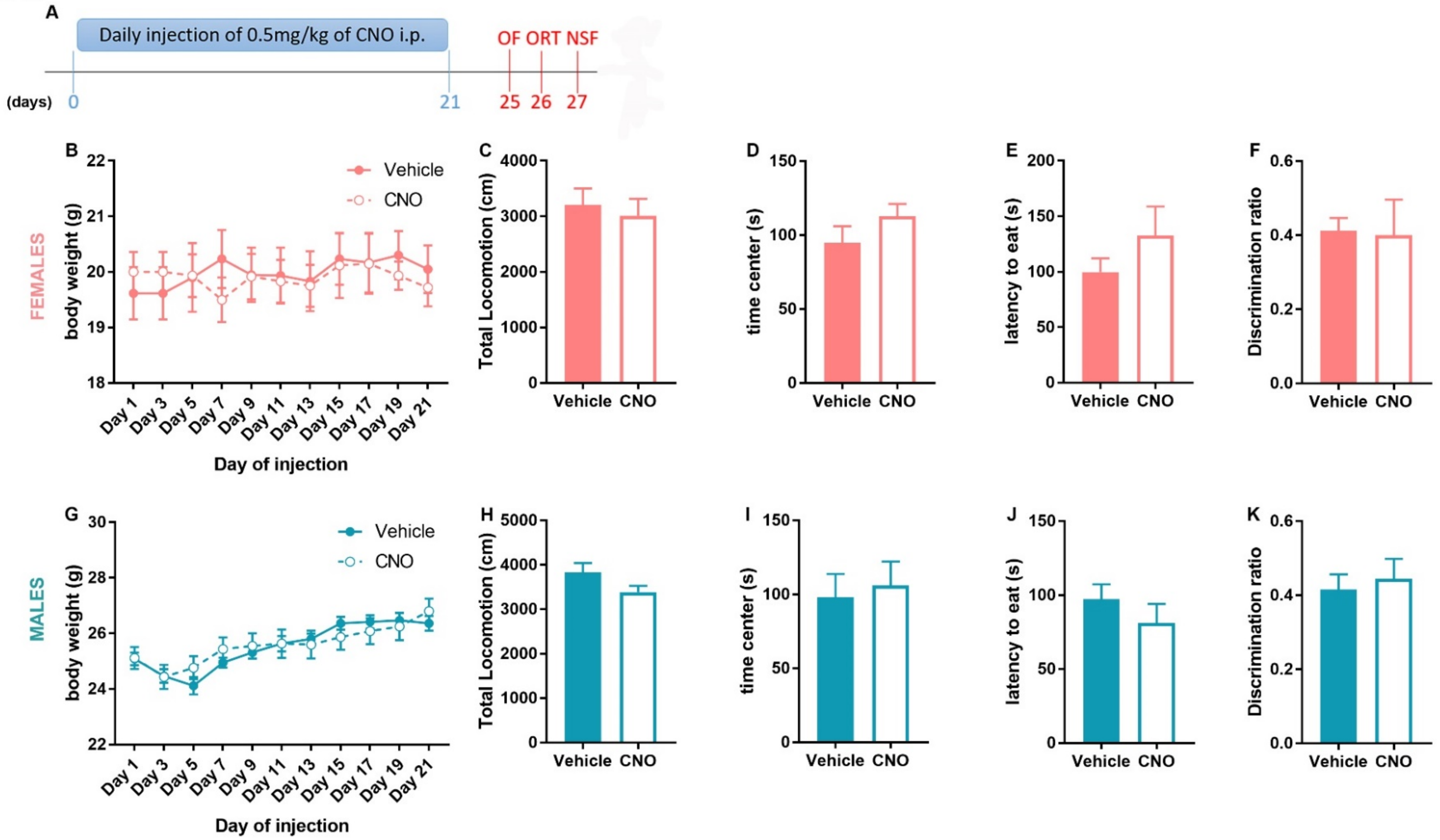

Figure S1: Chronic injection of CNO in male and female C57Bl/6 mice does not affect general health or behaviors. (**A**) Experimental scheme of the timeline followed. Mice were injected daily with 0.5mg/kg of CNO over the course of 21 days. Body weight was recorded throughout the injection period, and behaviors were testes four days after the last injection. No significant effect of CNO was observed. In all figures, summary data are represented as mean ± SEM. *N* = 6 per group per sex. CNO: clozapine-N-oxide; OF: open field; ORT: object recognition test; NSF: novelty-suppressed feeding test.

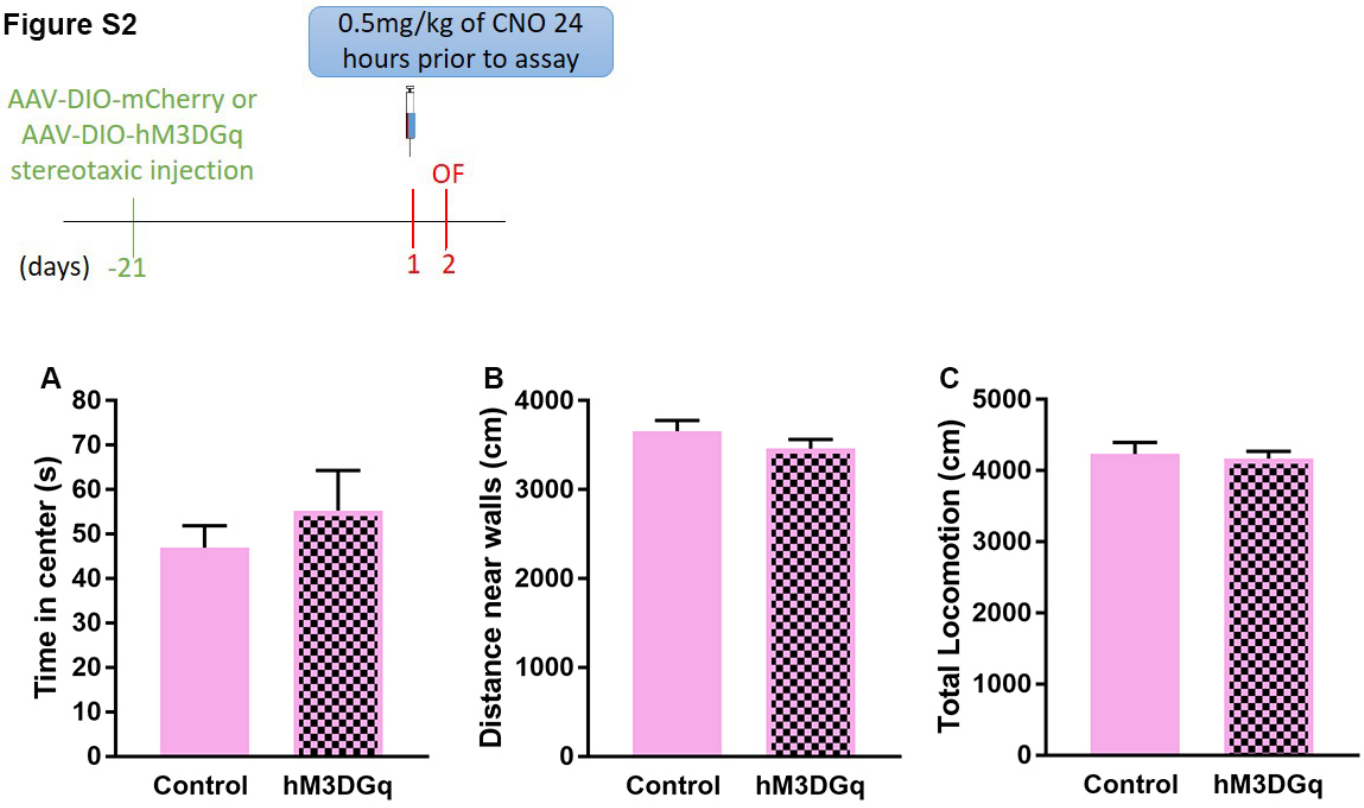

Figure S2: Prolonged effects of an acute chemogenetic activation of PV⁺ neurons in the mPFC of PV:Cre female mice. (Top) Mice injected with the control (AAV-DIO-mCherry) or the AAV-DIO-hM3DGq virus received one injection of CNO 24 hours prior to the OF. **(A-C)** Acute activation of mPFC PV⁺ neurons did not impact behaviors in the novel arena 24 hours later. N = 3 AAV-DIO-mCherry- and N=5 AAV-DIO-hM3DGq-injected female mice. OF: open field. Data were analyzed using a t-test ($p>0.05$ for all variables).
